# Supplementary figures and images for: Inhibition of polo-like kinase 1 (PLK1) facilitates reactivation of gamma-herpesviruses and their elimination
Source: PLoS Pathog. 2021 Jul 23;17(7):e1009764. doi: 10.1371/journal.ppat.1009764 (PMC8336821; doi:10.1371/journal.ppat.1009764)

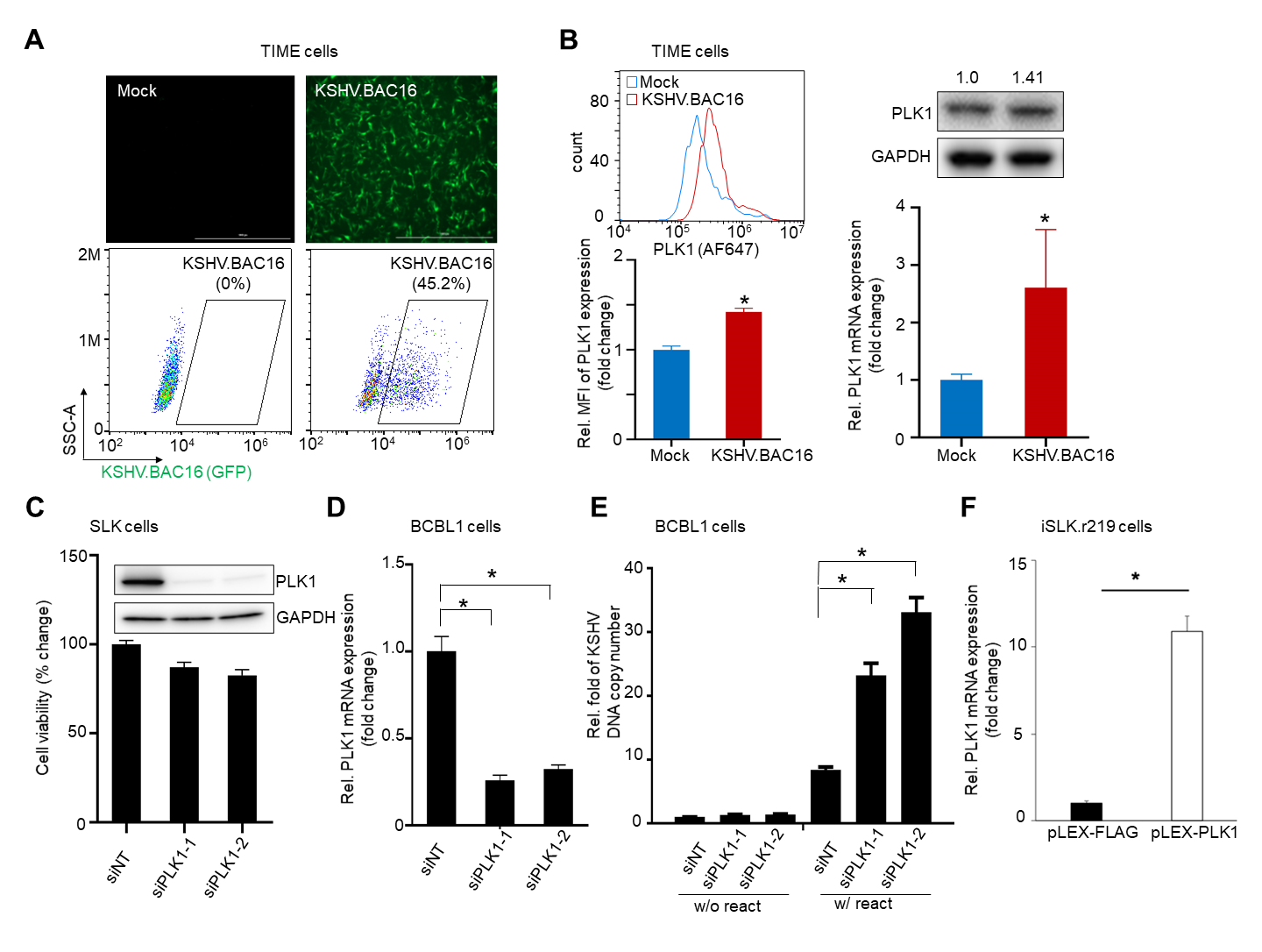

Supplement: S1 Fig — (A) TIME cells were de novo infected with KSHV.BAC16 viruses collected from supernatants of Dox-treated iSLK.BAC16 cells. At 48h post-of-infection, GFP+ cells were analyzed by fluroscence imaging or flow cytometry. (B) Cells in (A) were subjected to immunofluorescence assays of PLK1 and analzyed by flow cytometry. Mean fluorescence intensity (MFI) of PLK1 in these cells was measured. Alternatively, cells in (A) were also subjectd to PLK1 protein immunoblotting and mRNA RT-qPCR assays. Results were presented as mean ± SD (* p<0.05; two-tailed paired Student t-test). (C) SLK cells were transiently transfected with the indicated siRNAs (siNT, siPLK1-1, siPLK1-2), and subjected to PLK1 protein immunoblotting and cell viability assays. (D) BCBL1 cells were transiently transfected with the indicated siRNAs (siNT, siPLK1-1, siPLK1-2), and analyzed for PLK1 knockdown by RT-qPCR assays. (E) Cells in (D) were treated with TPA/NaB or mock, and supernatants were harvested and analyzed for KSHV viral DNA copy number by qPCR assays using primers targeting ORF73/LANA (KSHV). The standard curve was prepared by using the pA3M-LANA plasmid. The viral DNA copy number was calculated from the standard curve and normalized to cells. Results were presented as mean ± SD (* p<0.05; two-tailed paired Student t-test). (F) mRNA level of PLK1 in iSLK.r219 cells transiently transfected with the pLEX-FLAG or pLEX-PLK1 vector was measured by RT-qPCR. (* p<0.05; two-tailed paired Student t-test). (TIF) [file ppat.1009764.s001.tif]

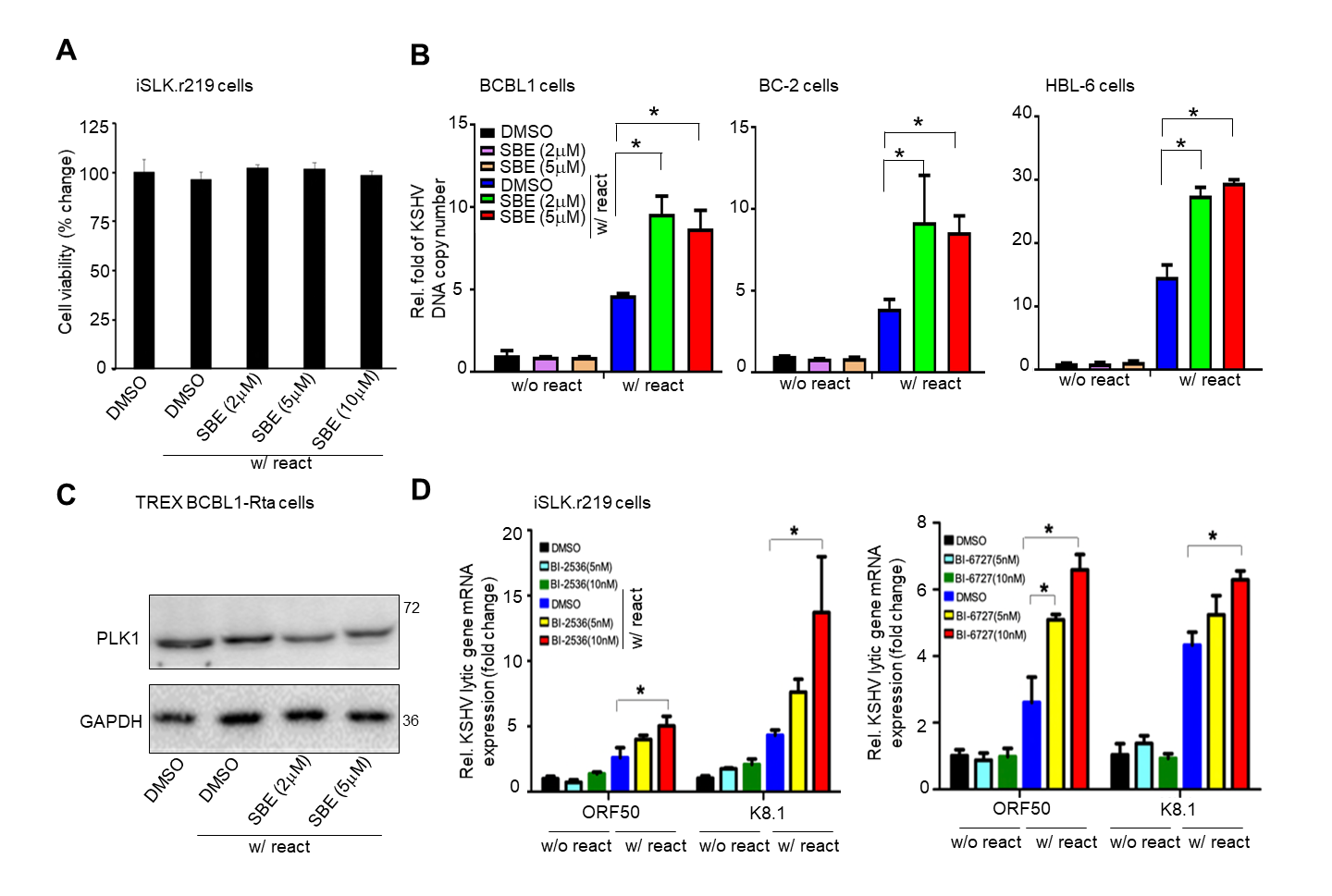

Supplement: S2 Fig — (A) Cell viability of iSLK.r219 cells treated with SBE at the increasing dose as well as Dox was measured by ATP-based assay. (B) BCBL1, BC-2, and HBL-6 cells were treated with SBE in the presence or absence of TPA/NaB, and supernatants were harvested and analyzed for KSHV viral DNA copy number by qPCR assays using primers targeting ORF73/LANA (KSHV). The standard curve was prepared by using the pA3M-LANA plasmid. The viral DNA copy number was calculated from the standard curve and normalized to cells. Results were presented as mean ± SD (* p<0.05; two-tailed paired Student t-test). (C) TREx BCBL1-Rta cells treated with SBE in the presence or absence of Dox was subjected to PLK1 protein immunoblotting assays. (D) mRNA level of KSHV lytic genes (ORF50, K8.1) in iSLK.r219 cells treated with BI-2536 or BI-6727 in the presence or absence of Dox was analyzed by qPCR and normalized to GAPDH. (* p<0.05; two-tailed paired Student t-test). (TIF) [file ppat.1009764.s002.tif]

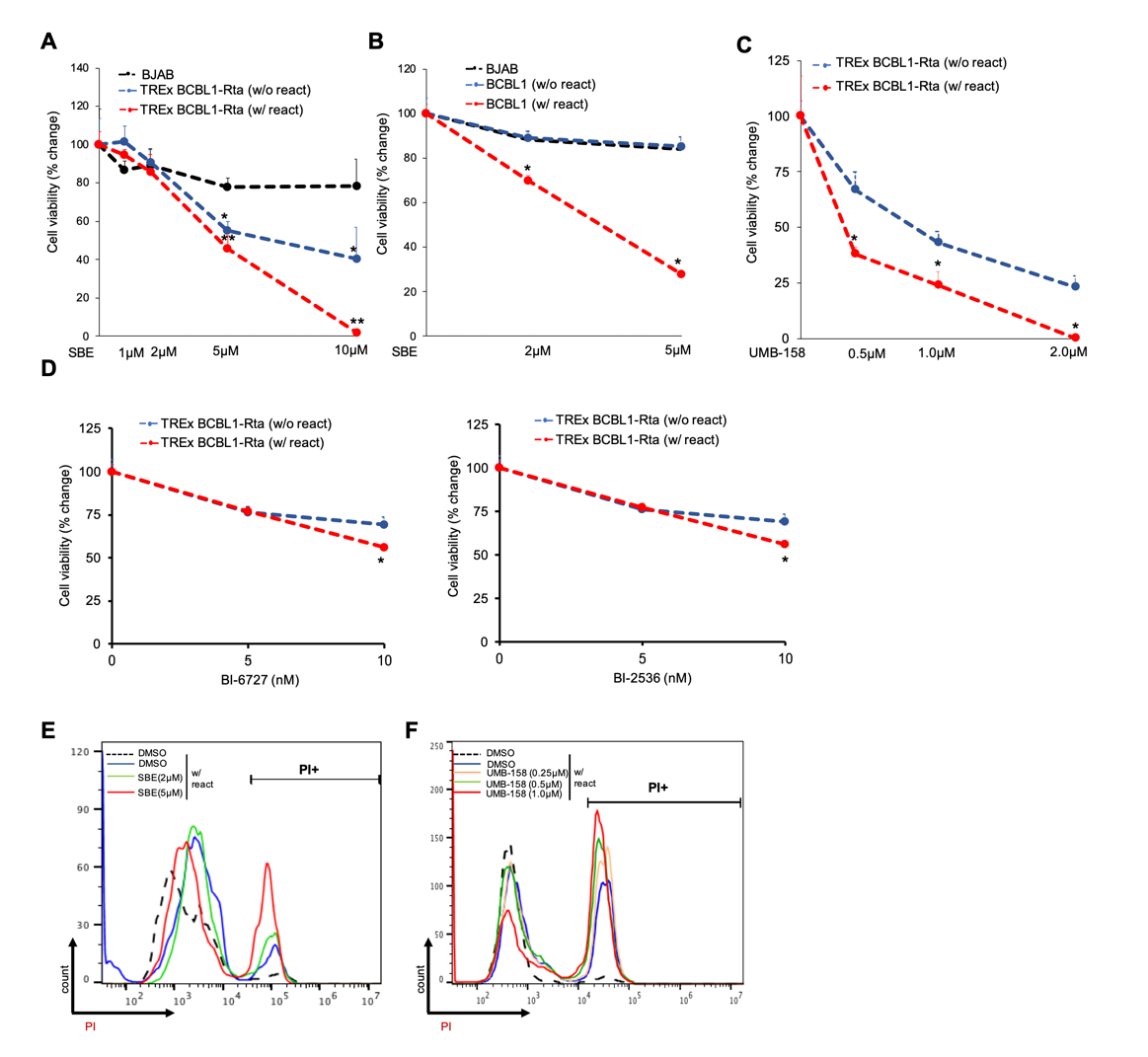

Supplement: S3 Fig — (A, B) Cell viability of TREx BCBL1-Rta (A) or BCBL (B) cells treated with SBE with or without Dox was measured by ATP-based assay. In parallel, cell viability of KSHV/EBV-negative BJAB cells treated with SBE alone was also measured. (C) Cell viability of TREx BCBL1-Rta cells treated with UMB-158 with or without Dox was measured. (* p<0.05, **p<0.01; two-tailed paired Student t-test). (D) Cell viability of TREx BCBL1-Rta cells treated with BI-6727 or BI-2536 in the presence or absence of Dox was measured by ATP-based assay. (* p<0.05; two-tailed paired Student t-test). (E, F) TREx BCBL1-Rta cells were treated with SBE (E), UMB-158 (F), or DMSO, and induced with Dox. Percentage of necrotic cells was analyzed by flow cytometry of PI-stained cells using Vybrant Apoptosis Assay Kit (Thermo Fisher),. (TIF) [file ppat.1009764.s003.tif]

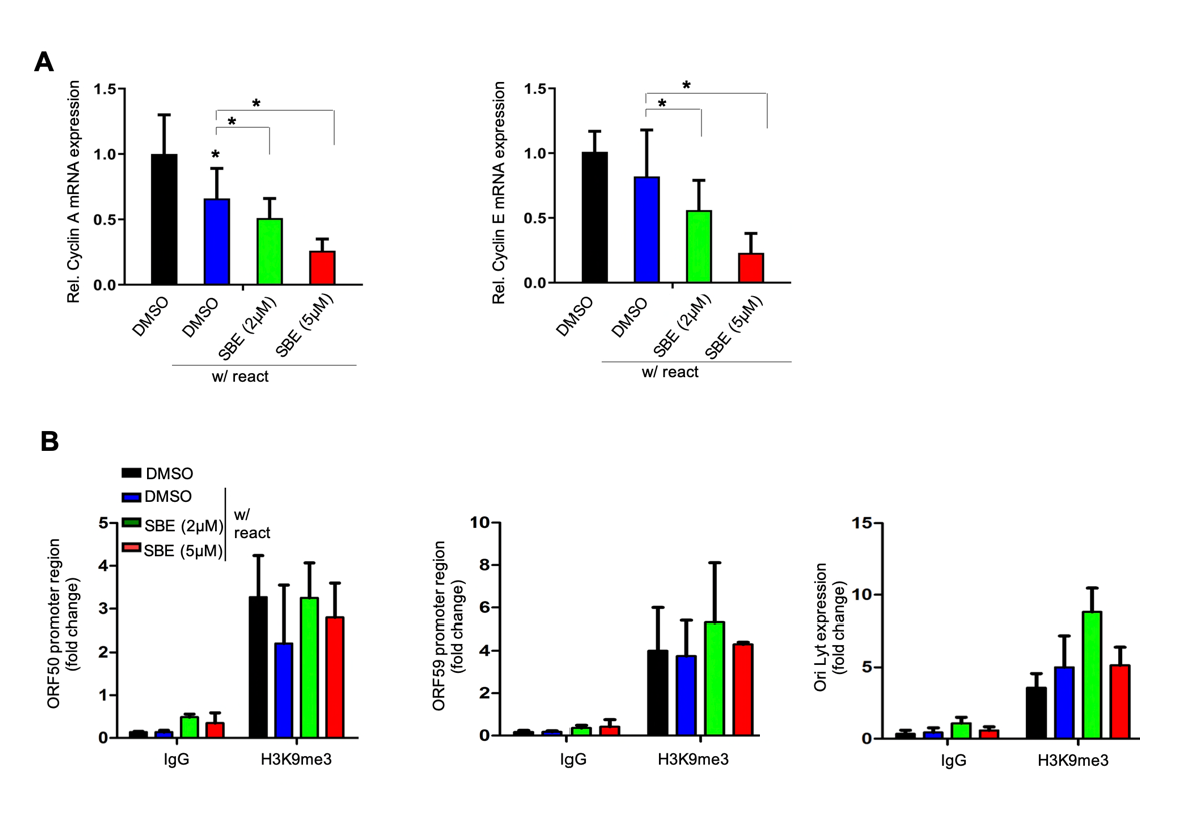

Supplement: S4 Fig — (A) mRNA level of cell-cycle genes, cyclin A and cyclin E, in TREx BCBL1-Rta cells treated with SBE or DMSO in the presence of Dox was measured by RT-qPCR and normalized to GAPDH. (B) TREx BCBL1-Rta cells were treated with SBE or DMSO, and Dox to induce KSHV reactivation. Cell lysates were prepared and subjected to ChIP assay by using antibodies against H3K9me3 or a mouse IgG. Precipitated DNA samples were further analyzed by qPCR by using primers targeting promoter region of KSHV lytic genes (ORF50, ORF59) and OriLyt, and normalized to IgG control. (* p<0.05; two-tailed paired Student t-test). (TIF) [file ppat.1009764.s004.tif]

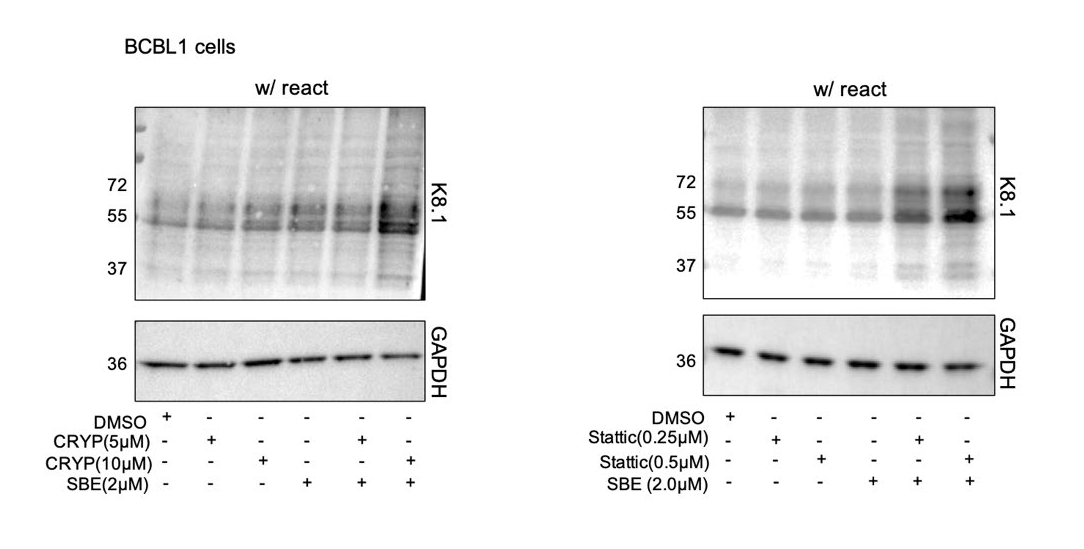

Supplement: S5 Fig — Protein level of KSHV K8.1 in BCBL1 cells treated with CRYP or stattic in the presence or absence of SBE was measured by immunoblotting. GAPDH was used as a loading control. (TIF) [file ppat.1009764.s005.tif]

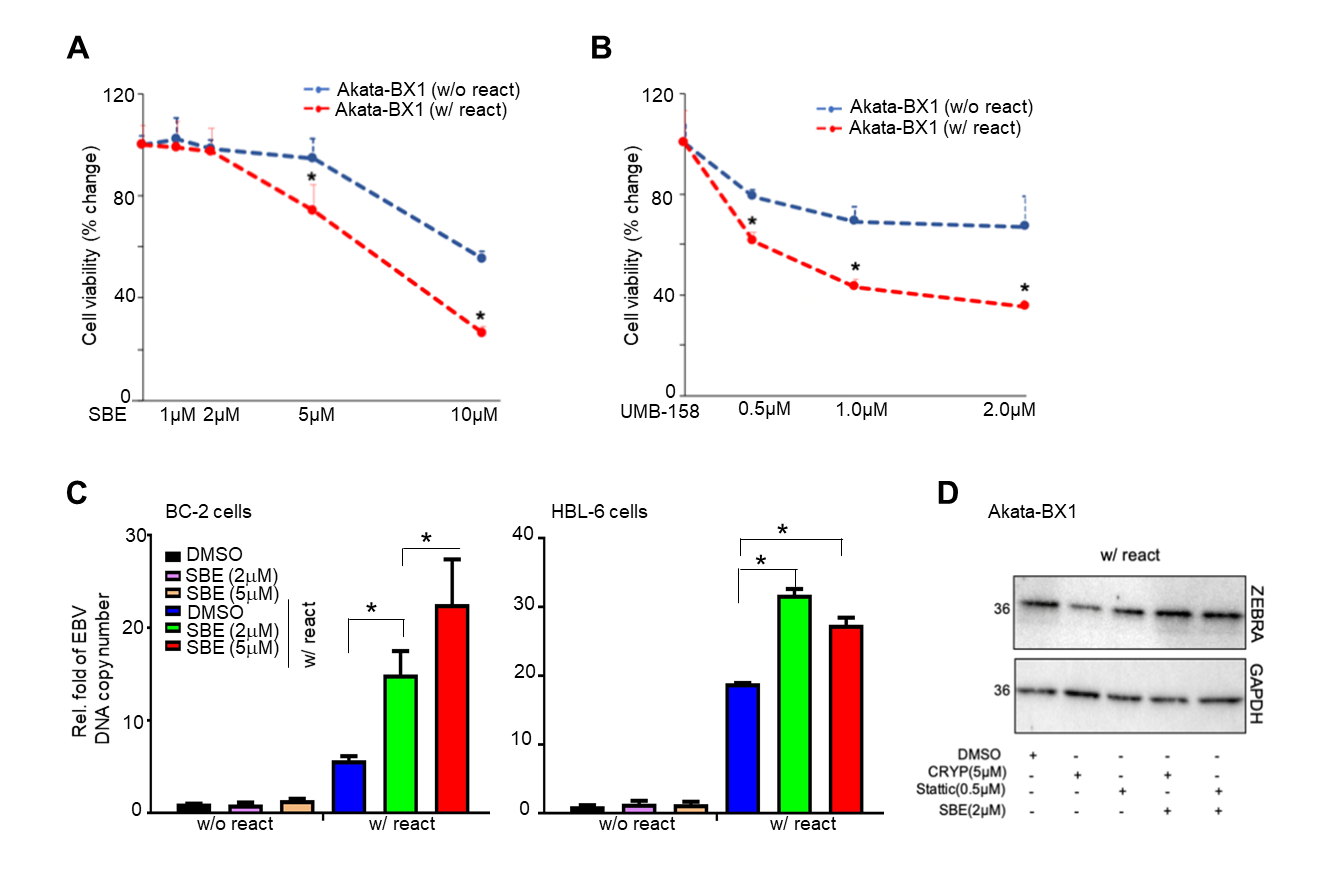

Supplement: S6 Fig — (A, B) Cell viability of Akata-BX1 cells treated with SBE (A) or UMB-158 (B) at the increasing dose with or without hIgG induction was measured by ATP-based assay. (* p<0.05, treatment vs control; two-tailed paired Student t-test). (C) BC-2 and HBL-6 cells were treated with SBE in the presence or absence of TPA/NaB, and supernatants were harvested and analyzed for EBV viral DNA copy number by qPCR assays using primers targeting EBNA1 (EBV). The standard curve was prepared by using the MSCV-N EBNA1 plasmid. The viral DNA copy number was calculated from the standard curve and normalized to cells. Results were presented as mean ± SD (* p<0.05; two-tailed paired Student t-test). (D) Protein level of EBV ZEBRA in Akata-BX1 cells treated with CRYP or stattic in the presence or absence of SBE was measured by immunoblotting. GAPDH was used as a loading control. (TIF) [file ppat.1009764.s006.tif]

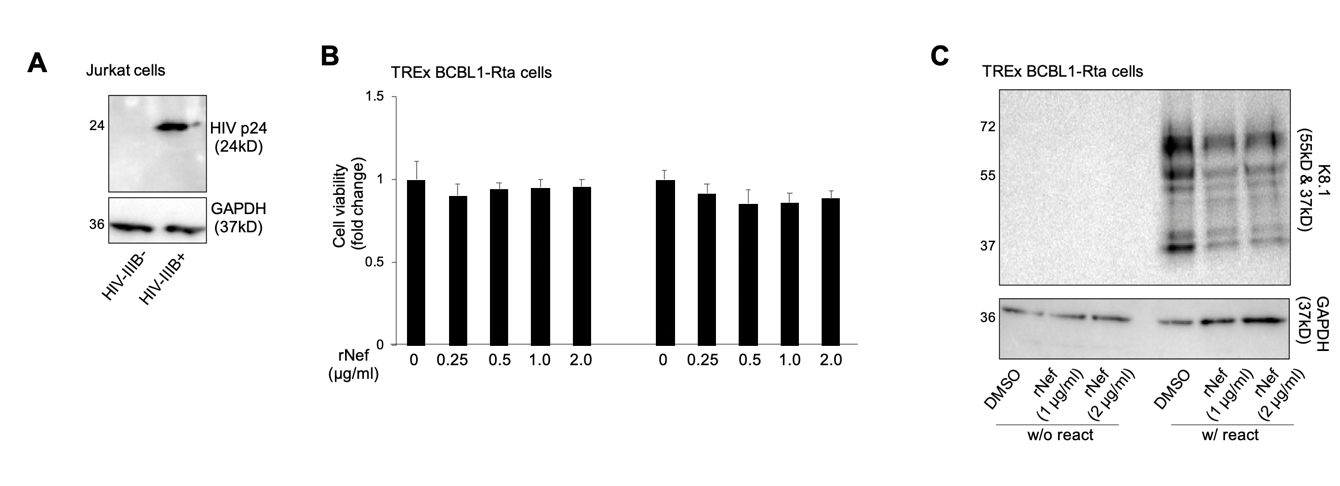

Supplement: S7 Fig — (A) HIV-1 IIIB infection in Jurkat cells was confirmed by immunoblotting of HIV p24 protein. (B) Cell viability of TREx BCBL1-Rta cells treated with rNef protein in the presence or absence of Dox was measured by ATP-based assay. (C) Protein level of KSHV K8.1 in above cells (B) was measured by immunoblotting. (TIF) [file ppat.1009764.s007.tif]

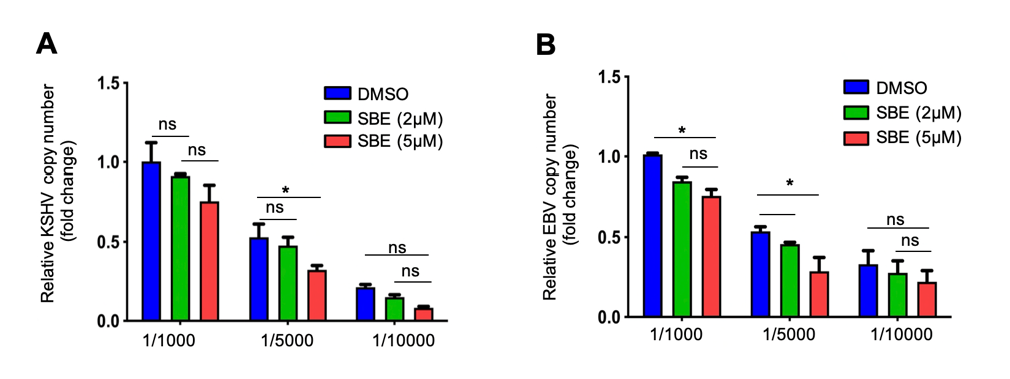

Supplement: S8 Fig — A serial dilution of TREx BCBL1-Rta (A) or Akata-BX1 (B) cells within KSHV/EBV-negative BJAB cells was prepared, followed by treatment of SBE alone or DMSO. Copy number of viral DNA genome was measured by qPCR using primers that target ORF73/LANA (KSHV) or EBNA1 (EBV) respectively, and normalized to GAPDH (* p<0.05; two-way ANOVA). (TIF) [file ppat.1009764.s008.tif]
