# Supplementary material for: Inhibition of polo-like kinase 1 (PLK1) facilitates reactivation of gamma-herpesviruses and their elimination
Source: PLoS Pathog. 2021 Jul 23;17(7):e1009764. doi: 10.1371/journal.ppat.1009764 (PMC8336821; doi:10.1371/journal.ppat.1009764)
Supplement: S1 Table — Primers used for qPCR assays in this study. (PDF) [file ppat.1009764.s009.pdf]

qPCR primers

Gene	Forward (5' to 3')	Reverse (5' to 3')
EBV_BZLF1	AATGCCGGGCCAAGTTTAAGCAAC	TTGGGCACATCTGCTTCAACAGGA
EBV_BRLF1	TGGCTTGGAAGACTTTCTGAGGCT	AATCTCCACACTCCCGGCTGTAAA
EBV_BMRF1	ATACGGTCAGTCCATCTCCT	CACTTTCTTGGGGTGCTT
EBV_BALF5	GCGGCCCCGGAGTTGTTA	CGTGGCCGTGGATCATTATTTC
EBV_BNRF1	GCAAACATACAGGAGGAAAG	CAGCAGGTTCTCAGCAATC
EBV_BLLF1	GCCTTGGAGAATATAACCTTG	CATTACTGTCTCGGGTCTTGG
EBV_BcLF1	GTGGATCAGGCCGTTATTGA	CCTCAAACCCGTGGATCATA
EBV_EBNA1	TGAGTCGTCTCCCCTTTGGA	CCTTAGCGGGCCAGGTTGTG
KSHV_ORF50	CCTTCGGCCCGGGGTCT	CGGTGGCAGTTGCGTATACTCT
KSHV_K8	CCTGGACGCTCTCTCACACA	GGATCTGCGAGTTGGAAGCT
KSHV_ORF26	AGCCGAAAGGATTCCACCAT	TCCGTGTTGTCTACGTCCAG
KSHV_LANA	TTACCTCCACCGGCACTCTT	GGATGGGATGGAGGGATTG
EBNA_1	GCCGGTGTGTTCGTATATGG	CAAAACCTCAGCAAATATATGAG
EBNA_2	CCACAATGTCGTCTTACACC	ATAACAGACAATGGACTCCCT
PR-ORF50	ATGAAGATGTGGTAGAGCCA	TAGCGCCATCTCTGCCCCCC
PR-ORF59	CACACTTCCACCTCCCCTAA	CGCACAGAGAAATCACAGGA
OriLyt	CCTACATGGGCAGCTTGTCC	TGCTGCCGGGGCTCCTCGTT
PLK1	CCA ACA CCA CGA ACA CGA	GCC TCT TGT CTC TTA GAT TTG GTC
GAPDH	GCC TCT TGT CTC TTA GAT TTG GTC	TAG CAC TCA CCA TGT AGT TGA GGT
**shRNA sequence**		
PLK1 shRNA	GTT CTT TAC TTC TGG CTA TAT	
NT shRNA	CAC AAA CGC TCT CAT CGA CAA G	
